# Comprehensive Evaluation of Nuclear Factor-κΒ Expression Patterns in Non-Small Cell Lung Cancer

**DOI:** 10.1371/journal.pone.0132527

**Published:** 2015-07-06

**Authors:** Ioanna Giopanou, Ioannis Lilis, Vassilios Papaleonidopoulos, Antonia Marazioti, Magda Spella, Malamati Vreka, Helen Papadaki, Georgios T. Stathopoulos

**Affiliations:** 1 Laboratory for Molecular Respiratory Carcinogenesis, Department of Physiology, Faculty of Medicine, University of Patras, Rio, Achaia, Greece; 2 Department of Anatomy, Faculty of Medicine, University of Patras, Rio, Achaia, Greece; 3 Comprehensive Pneumology Center (CPC), University Hospital, Ludwig-Maximilians University and Helmholtz Zentrum München, Member of the German Center for Lung Research (DZL), Munich, Germany; H. Lee Moffitt Cancer Center & Research Institute, UNITED STATES

## Abstract

Nuclear factor (NF)-κB signalling is required for lung adenocarcinoma development in mice, and both of its subunits *Rel*A and *Rel*B were independently reported to be highly expressed in human non-small cell lung cancer (NSCLC). To comprehensively examine NF-κB expression in NSCLC, we analyzed serial sections of primary tumor samples from 77 well-documented patients (36 adenocarcinomas, 40 squamous cell carcinomas and 3 large cell carcinomas) for immunoreactivity of *Rel*A, *Rel*B, P50, and P52/P100. Tumor and intratumoral stroma areas were discriminated based on proliferating cell nuclear antigen immunoreactivity and inflammatory infiltration was assessed in intratumoral stroma areas. NF-κB immunoreactivity was quantified by intensity, extent, and nuclear localization and was cross-examined with tumor cell proliferation, inflammatory infiltration, and clinical-pathologic data. We found that the expression of the different NF-κB subunits was not concordant, warranting our integral approach. Overall, *Rel*A, *Rel*B, and P50 were expressed at higher levels compared with P52/P100. However, *Rel*A and P50 were predominantly expressed in intratumoral stroma, but *Rel*B in tumor cells. Importantly, tumor area *Rel*A expression was correlated with the intensity of inflammatory infiltration, whereas *Rel*B expression was identified in proliferating tumor cells. Using multiple logistic regression, we identified that tumor *Rel*B expression was an independent predictor of lymph node metastasis, and tumor P50 was an independent predictor of TNM6 stage IIB or higher, whereas tumor *Rel*A was an independent predictor of inflammatory infiltration. We conclude that pathologic studies of NF-κB expression in cancer should include multiple pathway components. Utilizing such an approach, we identified intriguing associations between distinct NF-κB subunits and clinical and pathologic features of NSCLC.

## Introduction

Lung cancer is one of the most common human cancers and the leading cancer killer worldwide [1]. Lung cancer consists of two main histologic types, small-cell lung carcinoma and non-small-cell lung carcinoma (NSCLC), with the latter accounting for more than 80% of new cases [2]. NSCLC represents a histologically diverse group of tumors that are subclassified into adenocarcinomas, squamous cell carcinomas, and large cell carcinomas, accounting for 40, 30, and 10% of new NSCLC cases, respectively [2]. Adenocarcinoma is the predominant NSCLC subtype with rising incidence [3].

The mammalian nuclear factor (NF)-κB pathway is comprised mainly of *Rel*A, c-*Rel*, *Rel*B, NF-κB1 (P105/P50), and NF-κB2 (P100/P52) [4]. These ubiquitous proteins form homo- or heterodimers that reside in the cytoplasm in an inactive form, bound to inhibitory proteins known as inhibitors of NF-κB (ΙκΒ). Upon stimulation, IκB undergo phosphorylation by multiple IκB kinases (IKK; α, β, γ, ε, and others), as well as ubiquitination and proteolytic degradation by the proteasome and other proteases, to release active NF-κB subunits for nuclear translocation and activation of target gene transcription [5, 6]. There are two main NF-κB activation pathways, the canonical (or classical) and non-canonical (or alternative), which are mediated by *Rel*A/P50 or c-*Rel*/P50 and *Rel*B/P52 dimers, respectively [4–7]. More recently, additional mechanisms of NF-κB activation were unveiled, including but not limited to inducible processing of P100 to P52 regulated by IKKα homodimers, further increasing the complexity of the system [8]. Canonical and/or alternative NF-κB activation occurs after cellular stimulation by cytokines, bacteria or viruses, endotoxins, oxidative stress, irradiation, etc, and can lead to cell differentiation, proliferation, inhibition of apoptosis, and/or inflammatory signalling [9].

NF-κB activation has been identified in several human cancers, including but not limited to lymphoma, colorectal tumors, esophageal and head and neck carcinoma, breast tumors, hepatocellular carcinoma, and prostate cancer [10–16]. However, studies have focused almost exclusively on the canonical NF-κB pathway, while the role of alternative NF-κB signalling in tumor promotion and progression remains obscure [17–19]. In human NSCLC, increasing nuclear *Rel*A immunoreactivity has been described with progressive disease [20]. Canonical NF-κB signalling has also been found to be paramount for lung tumor formation in mouse models of NSCLC induced by oncogenic *KRAS* ^*G12D*^ and/or loss of the tumor suppressors *Trp53* and *Gprc5a*, cigarette smoke exposure, and the tobacco carcinogen urethane [21–25]. However, other studies suggest that constitutive or tumor microenvironment-induced NF-κB activation in NSCLC is more complex and rests on more than one pathway [26–28].

Here we set out to test this using comprehensive analyses of multiple NF-κB subunit expression of 77 well-documented patients with NSCLC. For this purpose, we developed a semi-quantitative NF-κB subunit scoring system that takes into account both expression levels as well as nuclear localization. We consistently identified distinct NF-κB subunit expression patterns in tumor cells versus intratumoral stroma and found interesting relationships between these patterns and clinical-pathologic features of human NSCLC. Finally, we recapitulated some of these findings in two different mouse models of NSCLC, indicating that divergent NF-κB activation patterns are indeed at play in NSCLC and may have different functions.

## Materials and Methods

### Patients

Seventy seven archival formalin-fixed, paraffin-embedded diagnostic tissue samples of patients with NSCLC that underwent surgical resection with curative intent between 2001 and 2008 at the University Hospital of Patras were retrospectively enrolled in the study. Some of these patients had also been studied in a previous report from our Institution [28]. Patients, all Caucasian, 69 men and 8 women aged from 46 to 84 years, were carefully selected based on the availability of full clinical and pathologic data extracted from the primary pathology reports and patients’ files, including age, sex, histologic type, grade, and detailed pathologic stage. Patients selected included 34 patients with lung adenocarcinomas, 40 with squamous cell carcinomas, and three with large cell carcinomas. All patients were staged according to the sixth edition of the TNM classification for NSCLC, current at the time of their diagnosis [29]. The study’s observational protocol was conducted according to the Declaration of Helsinki, was approved by the Ethics Committee of the University Hospital of Patras, Greece, and all patients gave written informed consent.

### Mice

Experiments were approved a priori by the Veterinary Administration of Western Greece (Protocol Number: 276134/14873/2) and were conducted according to Directive 2010/63/EU (http://eur-lex.europa.eu/LexUriServ/ LexUriServ.do?uri=OJ:L:2010:276:0033:0079:EN:PDF). For mutant KRAS-driven lung tumorigenesis, *C57BL*/6 mice heterozygous for the loxP-STOP-loxP.*KRAS* ^G12D^ transgene were used, which express mutant KRAS in any somatic cell upon CRE-mediated recombination (hereafter referred to as *Lsk* mice; Jackson Laboratories, Bar Harbor, MN) [22]. For lung tumor induction, *Lsk* mice received recombinant adenoviral vectors encoding CRE recombinase (Ad-*Cre*; Baylor College of Medicine, Cell and Gene Therapy Center, Vector Development Lab, Houston, TX) intratracheally (5x10^8^ plaque-forming units in 50 μl phosphate-buffered saline) and were sacrificed after four months [30]. For chemical-induced lung tumorigenesis, *FVB* mice (Jackson Laboratories, Bar Harbor, MN) received the tobacco carcinogen urethane intraperitoneally (1g/Kg in 100 μl phosphate-buffered saline) and were sacrificed after 6 months [21, 31]. Mouse lungs were exsanguinated by 20 ml intracardiac saline infusion, were inflated with 10% neutral-buffered formalin using 20 cmH_2_O pressure, were fixed in % neutral-buffered formalin, and were embedded in paraffin.

### Immunohistochemistry

Human and mouse tissue blocks were cut into 4 μm-thick sections, which were subsequently deparaffinised by ethanol gradient, rehydrated, and boiled in heat-induced epitope antigen retrieval solution (0.1 M sodium citrate; pH = 6.0). Endogenous peroxidase activity was inhibited using 3% H_2_O_2_ and non-specific antibody-protein binding was prevented using 3% bovine-serum albumin-containing Tris-buffered saline. The following primary antibodies and dilutions were used overnight at 4°C: anti-P50 (sc-114 rabbit polyclonal IgG; 1/150; Santa Cruz Biotechnology, Santa Cruz, CA), anti P100/P52 (ab31409 rabbit polyclonal IgG; 1/150; Abcam, Cambridge, UK), anti-*Rel*A (sc-8008 mouse monoclonal IgG; 1/200; Santa Cruz), anti-*Rel*B (sc-226 rabbit polyclonal IgG; 1/400; Santa Cruz), and anti-proliferating cell nuclear antigen (PCNA; ab2426 rabbit polyclonal IgG; 1/2000; Abcam). Detection of primary antibodies was performed using a horse radish peroxidase-conjugated polymer according to the manufacturer’s instructions (EnVision; Dako, Glostrup, Denmark) and diaminobenzidine as the chromogenic substrate. Sections were counterstained with Harris’s hematoxylin, dehydrated, and then mounted. For isotype controls, the primary antibody was omitted. Normal tonsil tissue was employed as positive control. All antibodies were validated for immunohistochemistry using serial dilutions starting from 1/100 (except from PCNA where dilutions started at 1/1000) and different bovine serum albumin concentrations in Tris–buffered saline following or modifying the manufacturer’s instructions in order to maximize the signal to background ratio. Immunoreactivity was scored by three blinded to the case investigators (I.G., H.P., and G.T.S.) including one Hellenic board-certified pathologist (H.P.) and consensus was sought in ambiguous cases by co-observation. PCNA immunoreactivity was defined as the percentage of positive cells in tumor areas and was used to discriminate these (high percentage of immunoreactive cells) from intra-tumoral stroma areas (low percentage of immunoreactive cells). Sections were counted at high power (X400), and 5–8 fields were assessed randomly for tumor cells and for intratumoral stroma areas, respectively. One thousand cell nuclei were counted respectively, and the number of cells showing positive nuclear staining was recorded. NF-κΒ subunit immunoreactivity was scored as follows: the intensity (0: negative; 1: weak; 2: moderate; and 3:strong), extent (0: <10% of cells positive; 1: 10–35% of cells positive; 2: 35–70% of cells positive; and 3: >70% of cells positive), and distribution (0: negative; 1: cytoplasmic only; 2: cytoplasmic and nuclear; and 3: nuclear only) of immunoreactivity were scored separately for each NF-κΒ subunit for the sample overall, as well as separately for tumor cells and intratumoral stroma of each tumor, and were combined into subunit-specific overall, tumor, and stroma scores using the formula: NF-κΒ subunit score = (intensity + extent) * distribution. NF-κΒ subunit scores were further categorized into low (0–4), intermediate (5–6), and high (7–18). Inflammatory infiltrates were determined semi-quantitatively using PCNA-stained and isotype control samples and was defined as low (<5%), moderate (5–20%), and high (>20%) depending on the percentage of leukocytes in intratumoral stroma areas. The latter were identified by morphology and lack of immunoreactivity for PCNA. Images were taken using an upright Axio Lab.A1 microscope connected to an AxioCam ERc 5s camera (Zeiss, Jena, Germany). For immunofluorescence, tissue sections were incubated simultaneously with anti-PCNA (either ab2426 rabbit polyclonal IgG; 1/100 dilution; Abcam for collocalization with *Rel*A or sc-56 mouse monclonal IgG; 1/100 dilution; Santa Cruz for collocalization with *Rel*B) and either anti-*Rel*A (sc-8008 mouse monoclonal IgG; 1/100 dilution; Santa Cruz) or anti-*Rel*B (sc-226 rabbit polyclonal IgG; 1/100 dilution; Santa Cruz) antibodies as above, followed by nuclear counterstaining with Hoechst 33258 (Molecular Probes, Eugene, OR). Detection of primary antibodies was performed using donkey anti-rabbit Alexa568-conjugated (A10042; 1/500 dilution; Invitrogen, Carlsbad, CA) and donkey anti-mouse Alexa488-conjugated (A21202; 1/500 dilution; Invitrogen, Carlsbad, CA) secondary antibodies. For isotype controls, either or both primary antibodies were omitted. Immunoreactivity was captured on a Zeiss Axio Observer.D1 inverted microscope connected to an AxioCam MRc 5 camera (Zeiss, Jena, Germany) and was co-registered using Fiji academic imaging freeware (http://fiji.sc/Fiji).

### Statistics

Probability values (*P*) less than 0.05 were considered significant. Human NF-κΒ subunit scores were not normally distributed, as tested by the Kolmogorov-Smirnov test (*P* < 0.05), and are shown in bar graphs as median with boxes indicating interquartile range and whiskers indicating 95% percentiles. Matched numerical NF-κΒ subunit scores of the same tumors were compared using Friedman’s tests followed by Dunn’s post-tests. Categorical NF-κΒ subunit scores were compared using χ^2^ tests followed by Fisher’s exact tests. Correlations between numerical NF-κΒ subunit scores and clinical-pathologic data were done using Spearman’s tests, whereby the overall threshold of statistical significance (*P* < 0.05) was adjusted to the total number of correlations examined (n = 153) by the Bonferroni method to *P* < 0.05/153 = 0.000327. Comparisons of numerical NF-κΒ subunit scores by inflammatory infiltration degree and clinical-pathologic categories were done using Wilcoxon signed rank tests or Kruskal-Wallis tests followed by Dunn’s post-tests, for two or multiple comparison groups, respectively. To determine whether numerical NF-κΒ subunit scores independently predict clinical-pathologic variables, the latter were dichotomized by their median and binary logistic regression analyses were performed using the backward Waldman method. For this, NF-κΒ subunit scores were used as the input (independent variables) and clinical-pathologic variables and inflammatory infiltration scores as the output (dependent variables). Mouse NF-κΒ subunit scores at progressive stages of carcinogenesis are presented as mean ± SD and were compared using two-way ANOVA followed by Bonferroni post-tests. Statistical analyses were performed using Prism v5.0.0 (GraphPad, San Diego, CA) and the Statistical Package for the Social Sciences v20 (IBM SPSS Statistics, Chicago, IL, USA).

## Results

### NF-κΒ subunit expression patterns in NSCLC

**Fig 1:**
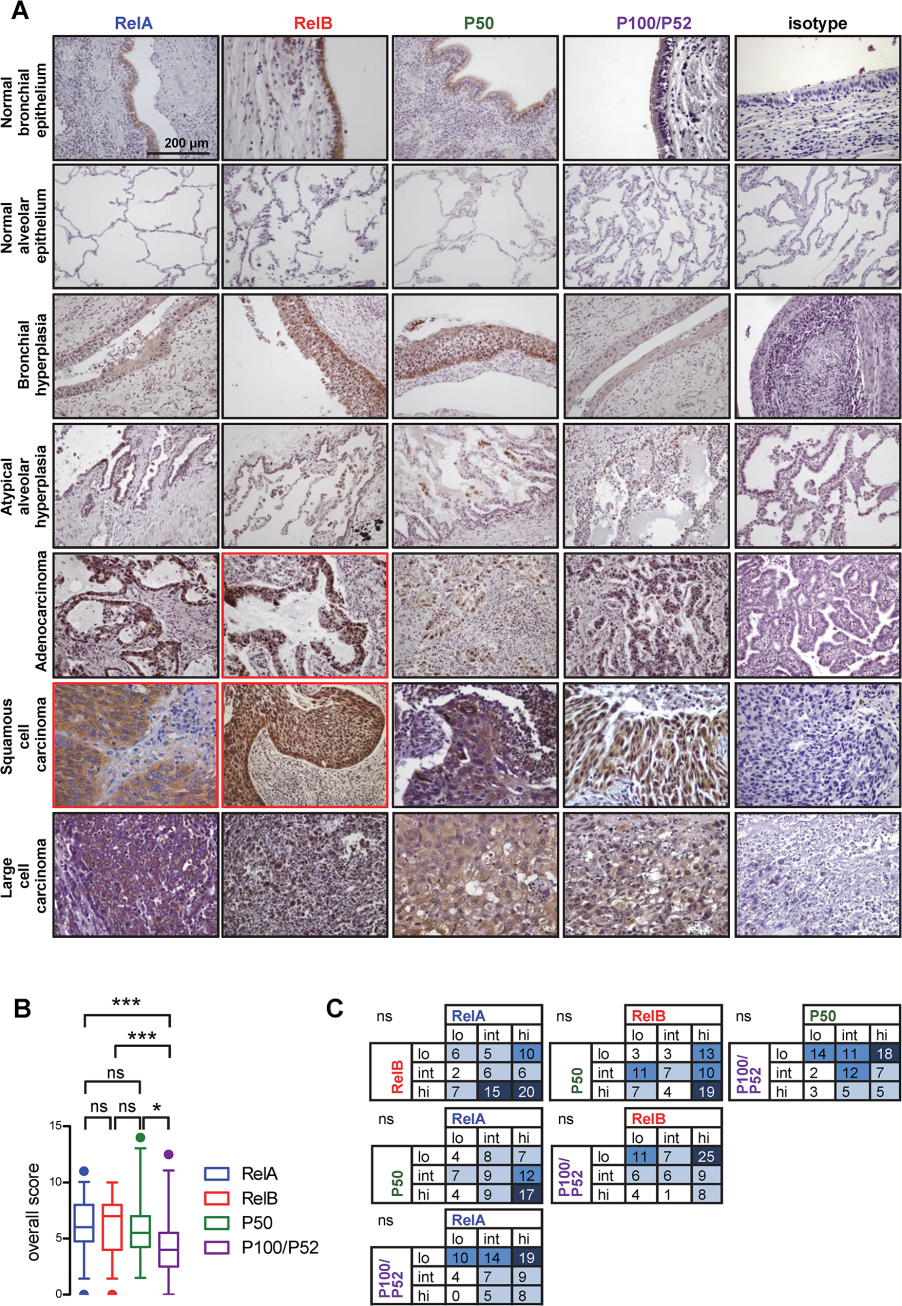
Immunohistochemical detection of NF-κB subunits in NSCLC, juxta-tumoral normal lung structures and preneoplastic lesions. **(A)** Representative images. Images in red frames representatively display differential NF-κB subunit expression in tumor and intratumoral stroma areas. **(B)** Overall scoring of NF-κB subunit expression levels. Data presented as median with boxes indicating interquartile range and whiskers indicating 95% percentiles. ns, * and ***: P > 0.05, P < 0.05, and P < 0.001 for indicated comparisons by Friedman’s test followed by Dunn’s post-tests. **(C)** Co-expression matrixes of categorical NF-κB subunit expression levels. For this, NF-κB scores from (B) were categorized into low (0–4), intermediate (5–6), and high (7–18). ns: P > 0.05 by χ^2^ tests followed by Fisher’s exact tests.

The clinical and pathologic features of the study patients are summarized in Table 1. The raw study results are appended to this article as S1 Table. We first examined NF-κΒ subunit expression in normal lung areas adjacent to our surgically resected tumor samples. In bronchial and alveolar epithelium, *Rel*A, P50 and P100/P52 exhibited low or moderate cytoplasmic immunoreactivity, while *Rel*B displayed higher expression levels. In juxta-tumoral bronchial and alveolar hyperplasias, immunoreactivity for all NF-κB subunits showed a stronger expression pattern, with *Rel*B showing both cytoplasmic and nuclear localization. In tumors, all NF-κB subunits were highly expressed relative to normal and hyperplastic areas, with *Rel*B scoring highest and showing the highest degree of nuclear localization. However, *Rel*A, *Rel*B, and P50 immunoreactivity was stronger compared with P100/P52 (Fig 1A and 1B). When NF-κB subunit scores were subdivided into low (0–4), intermediate (5–6), or high (7–18), no relationship was evident between the different subunits (Fig 1C). No statistically significant differences were noted between the different NSCLC histologic subtypes. However, statistically significant differences were observed in NF-κB subunit expression levels within each tumor: tumor areas displayed different expression patterns compared with intratumoral stroma areas (Fig 1A; representative panels in red frame). These results suggested that multiple NF-κΒ subunits are progressively overexpressed in NSCLC, showed that not all NF-κΒ subunits are expressed equally, and identified a discordance in the expression of multiple NF-κΒ subunits between tumor and intratumoral stromal areas, warranting further investigation.

**Table 1:** Clinical-pathologic features of 77 patients with NSCLC.

	adenocarcinoma	squamous cell carcinoma	large cell carcinoma
**Sex** (male/female; n)	29/5	37/3	2/1
**Age** (years; range)	46–84	48–81	47–54
**Differentiation** (n)			
None	0	0	2
Low	9	8	1
Low-intermediate	9	10	0
Intermediate	12	19	0
intermediate-high	2	2	0
High	2	1	0
**pathological TNM6 stage** (n)			
IA	3	3	0
IB	5	9	2
IIA	10	13	0
IIB	7	5	1
IIIA	8	10	0
IIIB	1	0	0

### Differential NF-κΒ expression in tumor and stromal compartments of NSCLC

**Fig 2:**
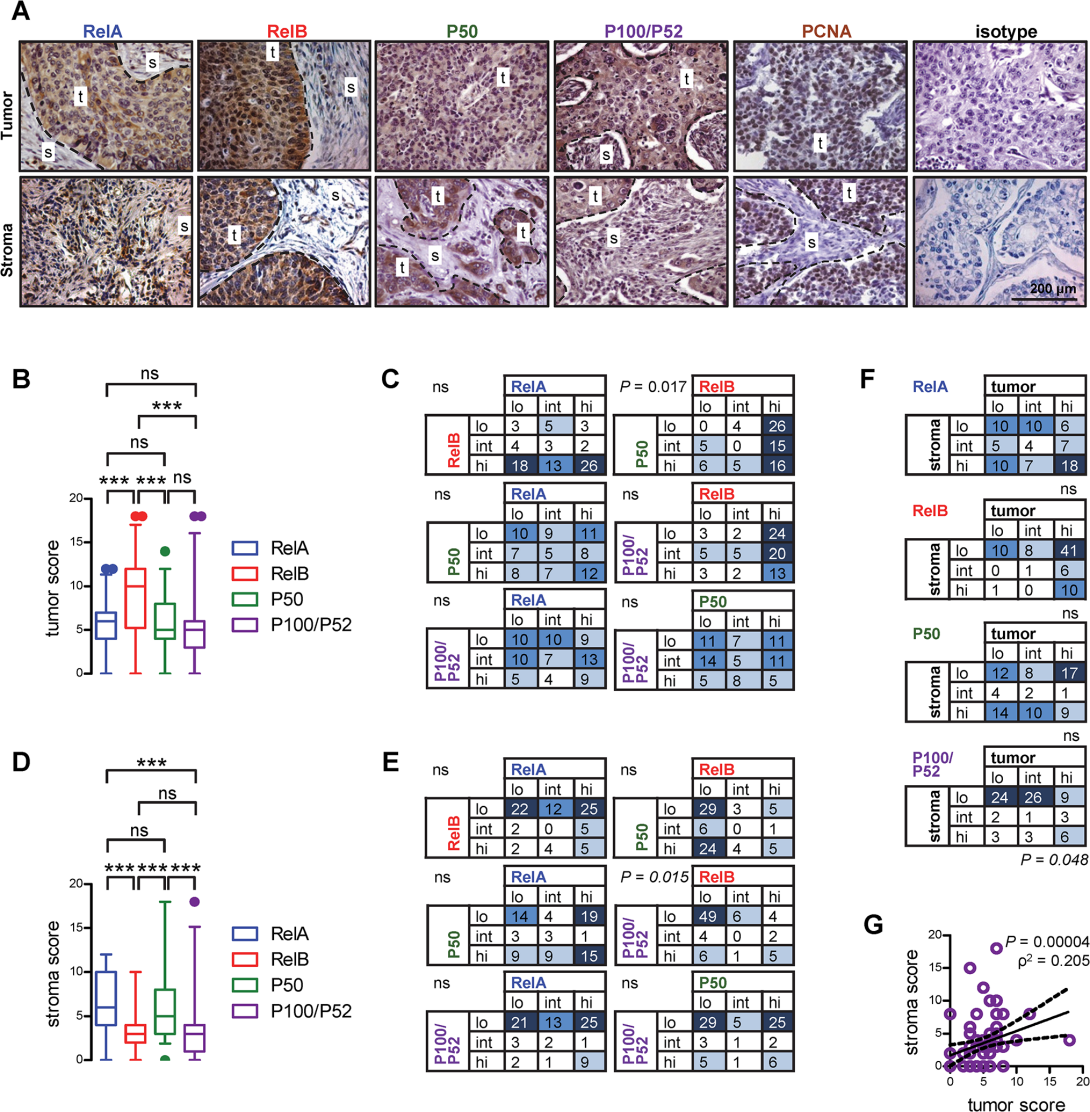
NF-κB subunit expression patterns in tumor versus intratumoral stroma areas. **(A)** Representative images. **(B, D)** Scoring of NF-κB subunit expression levels in tumor (B) and stroma (D) areas. Data presented as median with boxes indicating interquartile range and whiskers indicating 95% percentiles. ns and ***: P > 0.05 and P < 0.001 for indicated comparisons by Friedman’s test followed by Dunn’s post-tests. **(C, E)** Co-expression matrixes of categorical NF-κB subunit expression levels in tumor (C) and stroma (E) areas. For this, NF-κB scores from (B) and (D) were categorized into low (0–4), intermediate (5–6), and high (7–18). ns: P > 0.05 and P: probability values by χ^2^ tests followed by Fisher’s exact tests. **(F)** Co-expression matrixes of tumor versus stroma NF-κB subunit expression. ns: P > 0.05 and P: probability values by χ^2^ tests followed by Fisher’s exact tests. **(G)** Correlation of tumor and stroma P100/P52 expression scores. Shown are data points, linear regression line with 95% confidence interval, squared Spearman’s correlation coefficient, and probability value.

To study this in more detail, all samples were stained for PCNA, which clearly identified tumor areas (high abundance of PCNA+ cells) from the intratumoral stroma compartment (very low abundance of PCNA+ cells), and NF-κB subunit scoring was repeated separately for these two areas in serial sections of all patients (Fig 2A). These analyses revealed that *Rel*B was the subunit expressed the strongest in areas of tumor cells displaying a both nuclear and cytoplasmic expression pattern. On the other hand, the remaining subunits studied showed only moderate cytoplasmic expression in tumor areas (Fig 2A and 2B). When tumor compartment NF-κB subunit scores were subdivided into low (0–4), intermediate (5–6), or high (7–18) and compared within each tumor, *Rel*B and P50 showed significant discordance, with tumors with strong *Rel*B immunoreactivity exhibiting low P50 scores (Fig 2C). Surprisingly, evaluation exclusively of the intratumoral stroma yielded different results: here, *Rel*A and P50 subunits were most predominantly expressed in the nuclei of stromal cells. In stark contrast, *Rel*B and P100/P52 showed only weak cytoplasmic immunoreactivity (Fig 2A and 2D). When stroma NF-κB subunit scores were subdivided into low (0–4), intermediate (5–6), or high (7–18) and compared within each tumor, *Rel*B and P100/P52 showed significant concordance, with 49/77 tumors displaying simultaneously low scores for both subunits, likely reflecting the low expression levels of both proteins (Fig 2E). We finally examined the relationships between numerical and categorical tumor and stroma scores for each NF-κB subunit, finding that only P100/P52 expression in tumor and the related stroma were concordant and correlated (Fig 2F and 2G).

### Correlation of Rel subunit expression with cellular proliferation and inflammatory infiltration

**Fig 3:**
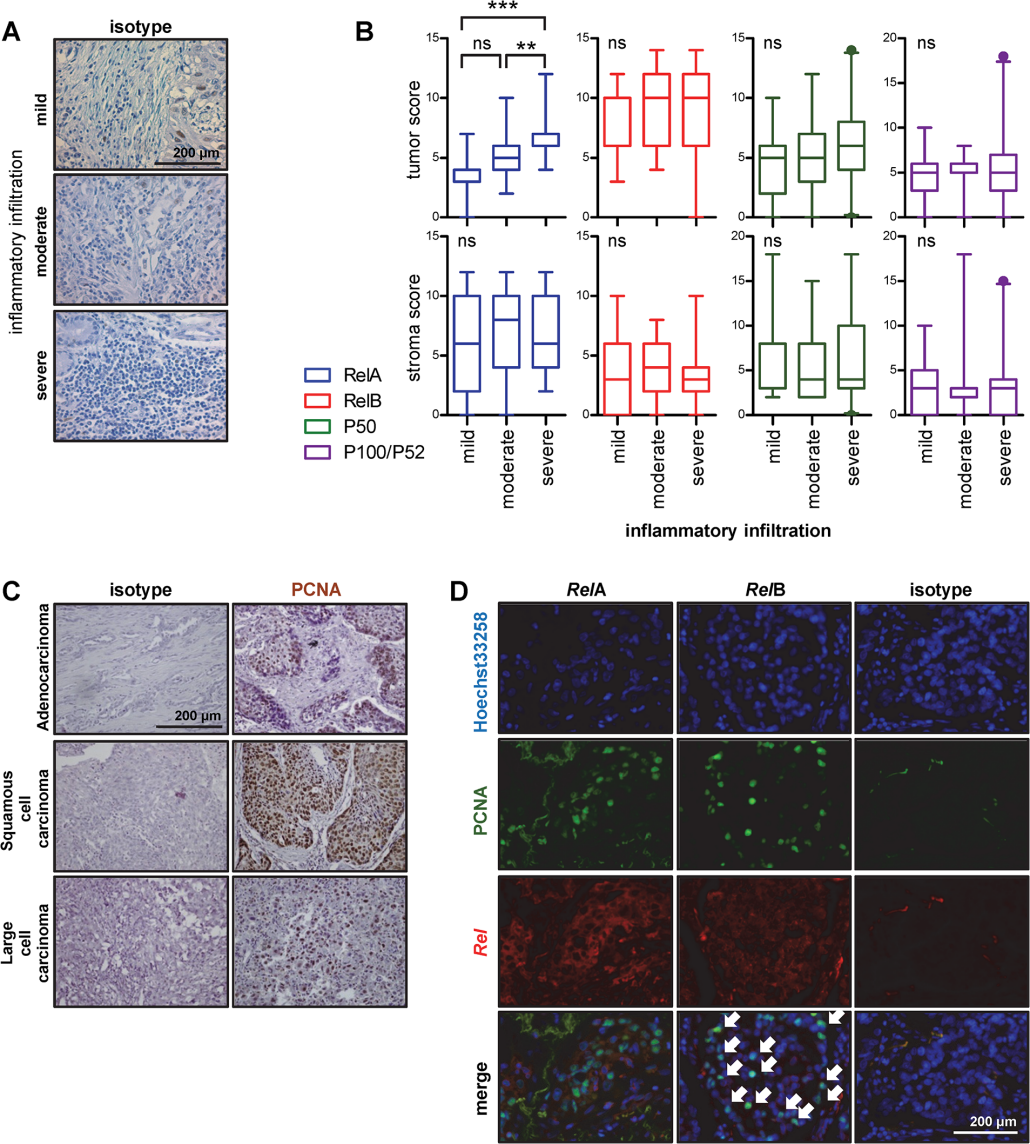
Association of NF-κB subunit expression with tumor-related inflammation and cellular proliferation in NSCLC. **(A)** Representative images of hematoxylin-stained samples showing different degrees of inflammatory infiltration of stroma areas. **(B)** NF-κB subunit expression scores of tumors with varying degrees of inflammatory infiltration. Data presented as median with boxes indicating interquartile range and whiskers indicating 95% percentiles. ns, **, and ***: P > 0.05, P < 0.01, and P < 0.001 for indicated comparisons by Kruskal-Wallis tests followed by Dunn’s post-tests. **(C)** Representative images of PCNA-stained NSCLC subtype samples. **(D)** Nuclear co-localization of PCNA immunoreactivity with *Rel*B (arrows), but not with *Rel*A, was identified using dual immunostaining of samples of 10 patients (representative images shown).

Since NF-κB is mechanistically implicated in the proliferation and inflammatory signalling of lung tumor cells in mouse models [21–24], we sought to determine the degree of cellular proliferation and inflammatory infiltration in our 77 patients with NSCLC. For this, serial sections were subjected to PCNA immunostaining as above, with simultaneous hematoxylin staining of isotype control sections, and the percentage of PCNA-positive cells in tumor areas was assessed as a measure of cellular proliferation rate. In addition, the percentage of tumor-infiltrating PCNA-negative inflammatory cells in stroma areas was determined for each patient (Fig 3A). These analyses identified that, although expression levels of *Rel*A in tumor areas were quite low, they were increased in tumors with high levels of inflammatory infiltration (Fig 3B). Moreover, although the percentage of PCNA-positive cells was not different between the different histologic subtypes (Fig 3C), and was not associated with the expression of any NF-κB subunit (data not shown), dual immunofluorescence for *Rel*A or *Rel*B combined with PCNA revealed that PCNA+ proliferating cells predominantly expressed *Rel*B in their nucleus (Fig 3D). These data indicated that tumor *Rel*A expression was associated with inflammatory infiltration and that tumor *Rel*B expression was linked with cellular proliferation within each tumor. Since *Rel*B expression has been found to be regulated by both the canonical as well as the alternative NF-κB pathways, we next sought to examine a possible correlation between *Rel*A expression in tumor or stroma compartments, and *Rel*B expression in tumor cells. However, no statistically significant correlation was found when the data were assessed either in a parametric or a non-parametric fashion, suggesting that tumor *Rel*A and *Rel*B expression occur independently in NSCLC.

### Association of NF-κΒ subunit expression with clinical and pathologic features of NSCLC

**Fig 4:**
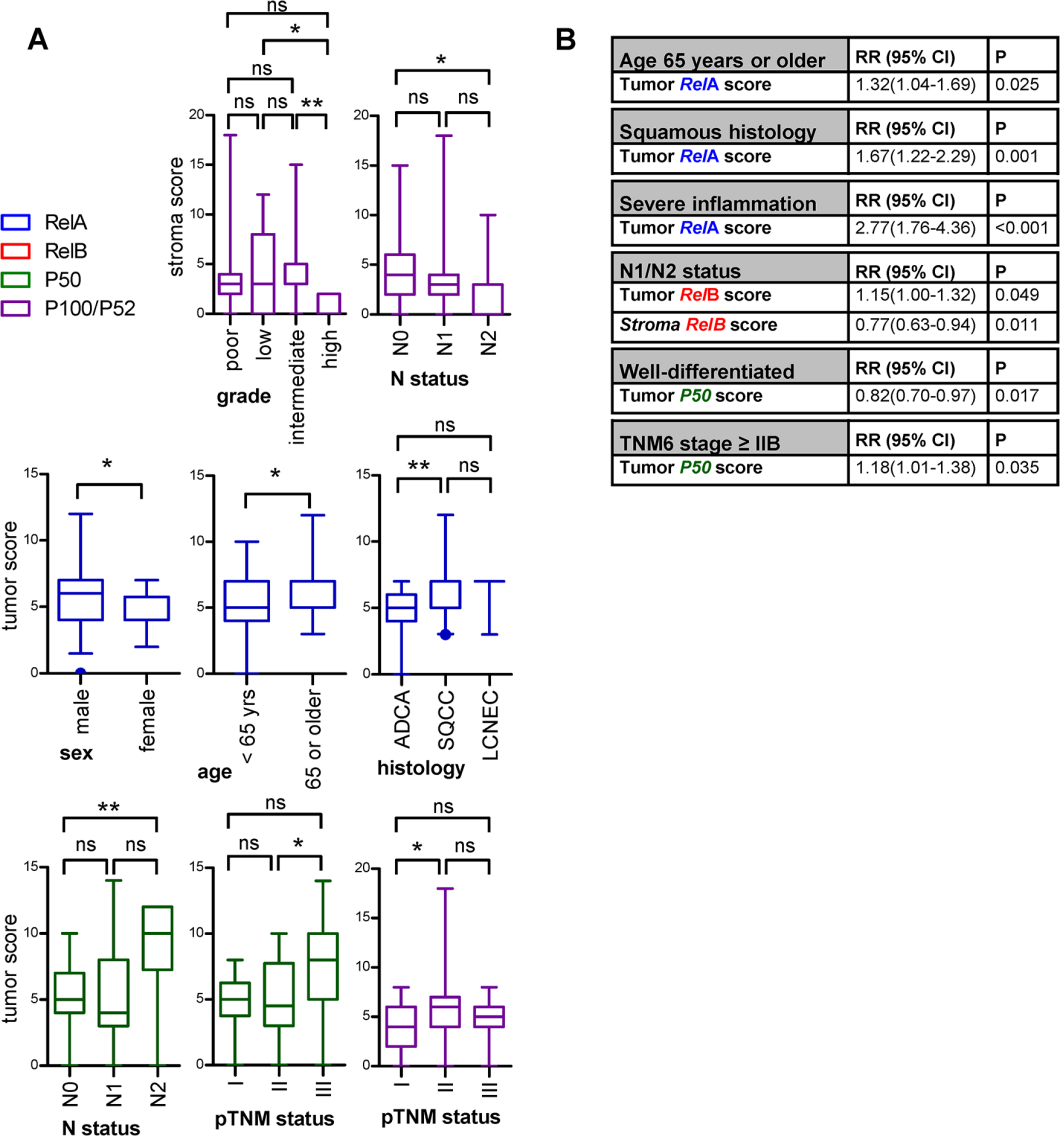
Association of NF-κB expression with clinical and pathologic parameters in 77 patients with NSCLC. **(A)** NF-κB expression levels subdivided by clinical and pathological parameters. Data presented as median with boxes indicating interquartile range and whiskers indicating 95% percentiles. ns, *, and **: P > 0.05, P < 0.05, and P < 0.0501 for indicated comparisons by Wilcoxon signed rank tests or Kruskal-Wallis tests followed by Dunn’s post-tests, for two or multiple comparison groups, respectively. **(B)** Results of binary logistic regression analyses using NF-κB subunit expression scores as the input (independent variables) and dichotomized clinical and pathologic parameters as the output (dependent variables). RR, risk ratios; CI, confidence intervals; P, probability values.

We next examined whether NF-κB subunit expression in tumor and stroma compartments is correlated with clinical and pathologic features of our patients with NSCLC. For this, correlation analyses were done, which revealed no significant associations (data not shown). NF-κB scores were further subdivided according to clinical and pathologic features and subgroups were compared (Fig 4A). The stroma score of P100/P52 was significantly lower in well-differentiated tumors, as compared with low or moderately differentiated ones. In addition, stromal P100/P52 expression was decreased in patients with N2 disease, as compared with patients without lymphatic involvement. No other significant differences were identified regarding the intratumoral stromal expression of any other subunit. When tumor area NF-κB scores were examined this way, *Rel*A was found to be expressed at higher levels in men compared with women, in patients aged 65 and over compared with younger ones, and in patients with squamous cell carcinoma compared with adenocarcinoma. Importantly, tumor P50 scores were elevated in patients with N2 and pTNM6 stage III disease compared with patients without lymphatic involvement and stage II disease, respectively. Finally, tumor P100/P52 expression was statistically significantly increased in patients with pTNM6 stage II disease compared with patients with stage I disease. In a third level of investigations, clinical and pathologic NSCLC features were dichotomized by their median and were entered as dependents into binary logistic regression analyses, using tumor and stromal NF-κB scores as the input (independent) variables (Fig 4B). These analyses revealed that tumor *Rel*A score was an independent predictor of age 65 or older, of squamous histology, and of inflammatory infiltration. Interestingly, tumor and stroma *Rel*B expression were identified as positive and negative predictors of nodal involvement, respectively. Finally, tumor P50 score was found to be a independent predictor of poor differentiation and pTNM6 stage IIB or higher. Hence distinct NF-κB subunit (*Rel*B and P50, but not *Rel*A) expression was associated with advanced disease, and distinct NF-κB subunits were linked with defined features of NSCLC, validating our integrated approach.

### NF-κΒ subunit expression in mouse models of NSCLC

**Fig 5:**
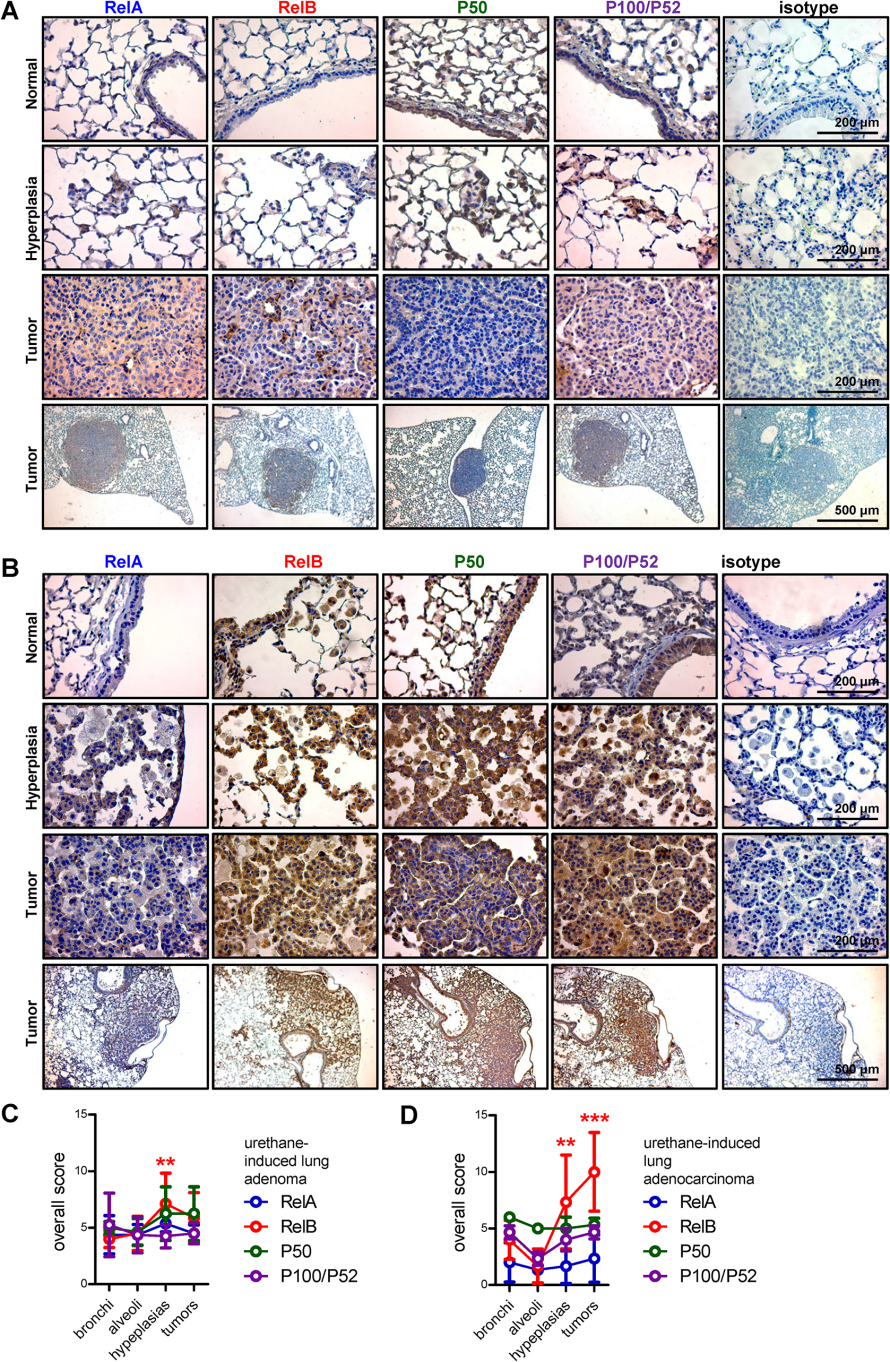
Immunohistochemical detection of NF-κB in mouse models of NSCLC. NF-κB subunit expression was assessed by immunohistochemistry in urethane-induced mouse lung adenomas **(A and C)** and mutant *KRAS*-induced lung adenocarcinomas **(B and D)**. **(A, B)** Representative images. **(C, D)** Overall scoring of NF-κB subunit expression levels from four mice per group. Data presented as mean ± SD. ** and ***: P < 0.01, and P < 0.001 for the indicated color-coded subunit compared with normal bronchial and alveolar epithelium by two-way ANOVA followed by Bonferroni post-tests. Non-significant comparisons are not indicated.

We finally sought to verify whether our findings could also be recapitulated in two different mouse models of human NSCLC, namely the model of urethane-induced pulmonary adenoma and of mutant *KRAS*-induced pulmonary adenocarcinoma. Analyses, done as for human samples, identified that *Rel*B was overexpressed in urethane-induced hyperplastic lesions, but not in adenomas. Moreover, *Rel*B was overexpressed in both mutant *KRAS*-induced hyperplastic lesions and adenocarcinomas (Fig 5). These findings were consistent with the human data showing focal overexpression of *Rel*B in tumor areas of human NSCLC (Fig 2).

## Discussion

**Fig 6:**
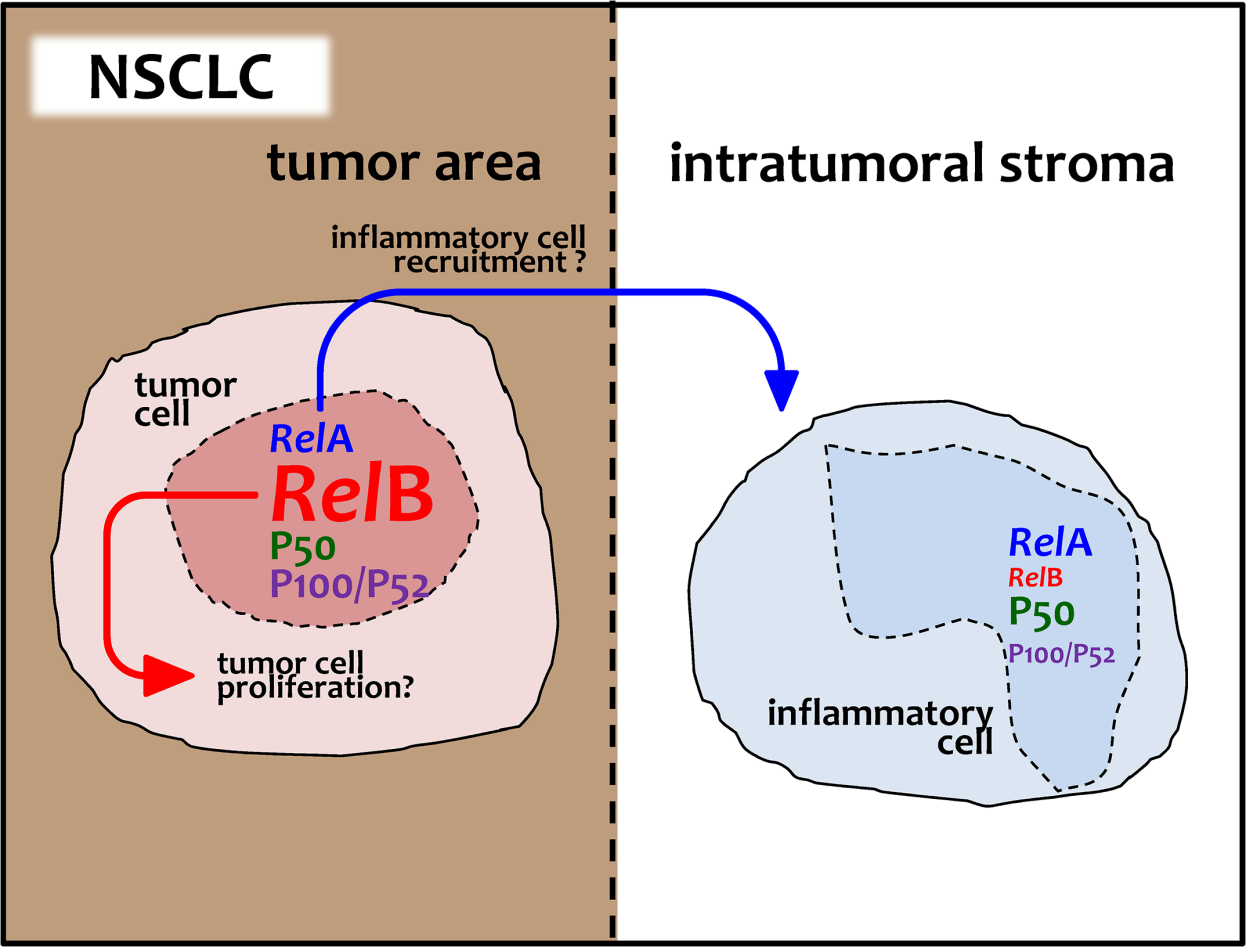
Schematic illustration of the main findings of the present study. NF-κB subunit expression levels in tumor and stroma cells of 77 patients with NSCLC are indicated by relative font size. Arrows indicate possible associations of *Rel* protein expression levels in NSCLC tumor cells with tumor-associated inflammation and cellular proliferation.

In the present study, we characterized the expression and the subcellular localization of several NF-κB subunits in human NSCLC and experimental models of the disease. We found different patterns of immunoreactivity for each of the subunits studied, justifying our integrated approach. In specific, *Rel*B was highly expressed in tumor cells, while *Rel*A and P50 in stromal cells. Interestingly, tumor *Rel*A expression was correlated with inflammatory infiltration and tumor *Rel*B expression was collocalized with proliferating tumor cell nuclei. These results were consistent with binary logistic regression analyses that revealed that tumor *Rel*A expression was an independent predictor of inflammatory infiltrates, while tumor *Rel*B immunoreactivity predicted more advanced disease with nodal involvement. Finally, this pattern of predominant *Rel*B expression in human NSCLC was recapitulated in mouse models of the disease. Collectively, our results indicate a multi-modal activation of NF-κB in NSCLC and possibly suggest divergent functions for *Rel*A and *Rel*B in driving paracrine induction of inflammation and cell-autonomous or autocrine promotion of cellular proliferation, respectively (Fig 6).

Although previous reports from various human cancers support a predominantly canonical NF-κB activation pathway mostly mediated by *Rel*A/P50 [32–37], recent data along with ours highlight the possibility for a non canonical NF-κB activation pattern in different cancers [38,39]. In 2005, Lessard and colleagues reported investigations on the expression of multiple NF-κB subunits in prostate cancer tissue arrays; they found nuclear subunit combinations such as *Rel*B–P100/P52 and *Rel*A–*Rel*B, introducing for the first time a different NF-κB pattern associated with the progression of the disease [39]. In addition, a more recent study of head and neck squamous cell carcinoma proposed a combined effect of both IKKα and IKKβ on the nuclear localization of canonical *Rel*A and alternative *Rel*B and P100/P52 subunits [40]. These studies, collectively with our results, suggest a possible alternative NF-κB activation pattern in malignant versus benign cells. This could imply altered intracellular and paracrine signalling from tumor cells in response to alternative NF-κB activation, since *Rel*A-P50 and *Rel*B–P100/P52 complexes bind to NF-κB binding sites of different promoters [41]. In addition, several studies correlate the nuclear membrane transporter chromosomal region maintenance/exportin1 protein (CRM1) with tumor progression in several types of cancers and CRM1 is known to export *Rel*A from the nucleus into the cytoplasm in ovarian cancer, a phenomenon which could explain the observed cytoplasmic localization of *Rel*A in the present and other studies [42–45].

Both *Rel*A and *Rel*B nuclear immunoreactivity have been independently identified in human NSCLC by two different groups [20, 28]. We report here a possible explanation for the different results: we identified for the first time that tumor and intratumoral stroma compartments display distinct NF-κB subunit expression patterns, predominantly signified by non-canonical and canonical subunits, respectively. This contrasting expression pattern could account for the divergent results, if intratumoral stromal areas were to be interpreted as reflecting tumor areas in terms of NF-κB subunit expression, and warrant careful discrimination of these distinct areas within each tumor when looking for NF-κB immunoreactivity. Moreover, we show how this can be readily done using PCNA immunostaining.

A large body of evidence supports that inflammation fuels the progression of bodily tumors, including lung cancer [46, 47]. In addition to promoting tumor cell proliferation, NF-κB is a cardinal mediator of innate immune responses in the lungs and other organs, by driving the expression of several genes involved in cell cycle progression and non-cell-autonomous inflammatory signalling [48]. Our findings suggest dual functions for canonical and alternative NF-κB pathway components in these two processes: *Rel*A expression in tumor areas was elevated in tumors with higher degrees of inflammation and independently predicted severe tumor-associated inflammation, while nuclear *Rel*B coincided with proliferating cells within each tumor. Hence, in NSCLC, *Rel*A may promote the expression of secretory genes encoding proinflammatory cytokines and chemokines responsible for attracting inflammatory cells to the tumor site, with possible pro-tumor or anti-tumor functions [48]. In turn, *Rel*B may facilitate the expression of cell cycle genes that promote cellular proliferation in a cell-autonomous fashion.

Studies in animal models are in accord with our findings and consistent with the hypothesis that non-canonical NF-κB activation pathways are at play in NSCLC. In particular, a previous study from our group identified that the proteasome (and hence canonical NF-κB pathway) inhibitor bortezomib dramatically enhanced instead of halting chemical-induced lung carcinogenesis in mice [49]. Bortezomib has also shown limited efficacy against human NSCLC [50]. Collectively, our data presented here and other reports from the literature indicate that non-canonical NF-κB transcriptional activity may exist in lung and other tumors.

In addition to triggering tumor-associated inflammation, NF-κΒ is positioned to critically impact tumor cell proliferation. We found that *Rel*B was expressed in dividing cells of NSCLC, suggesting that this NF-κΒ subunit may be linked with NSCLC cellular proliferation in an either positive or negative fashion. Although our study’s design does not allow for the deduction of functional conclusions on *Rel*B effects on NSCLC cell proliferation, a recent study of head and neck squamous carcinomas revealed that IKKα, cooperatively with IKKβ, promotes cellular growth as well as nuclear *Rel*B translocation in this tumor type, suggesting possible pro-tumor effects for *Rel*B [40]. In addition, Jacque and co-workers showed that *Rel*B exerts inhibitory effects on tumor cell proliferation by up-regulating tumor protein 53 (TP53) expression, which are lost upon the occurrence of *TP53* mutations or loss [51]. We speculate that *Rel*B may exert pivotal effects on tumor cell proliferation in our patients with NSCLC, since many patients with this tumor type have been consistently and repetitively shown to feature all kinds of *TP53* perturbations, including copy number alterations and point mutations, while a significant proportion retain the wild-type tumor suppressor. In any case, our results collectively with the literature warrant further investigation of *Rel*B functions in cancer.

The limitations of our study are not to be overlooked. First, the descriptive nature of our findings do not allow for functional implications prior to experimental validation. Second, since the majority of our study’s participants were male, our conclusions should be regarded as male-specific. In addition, the limited number of patients with large cell carcinoma does not allow for conclusions to be drawn for this tumor type.

In summary and despite their inherent limitations, our findings support that alternative modes of NF-κB activity are functional in human and murine NSCLC, in addition to canonical NF-κB activity. This non-canonical NF-κB activity may be responsible for the lack of efficacy of canonical NF-κB inhibitors against NSCLC and warrants further investigation.

## Supporting Information

S1 Table Raw data of present study. (XLSX)
